# Association of Low Levels of Soluble Low-Density Lipoprotein Receptor-Related Protein-1 and COVID-19 Outcomes During the First Wave of Pandemic

**DOI:** 10.7759/cureus.37254

**Published:** 2023-04-07

**Authors:** Mehmet Agirbasli, Rabia Korkmaz, Ferruh K Isman

**Affiliations:** 1 Department of Cardiology, İstanbul Medeniyet University, Faculty of Medicine, Istanbul, TUR; 2 Department of Medical Biochemistry, Istanbul Medeniyet University, School of Medicine, Istanbul, TUR; 3 Department of Medical Biochemistry, Istanbul Medeniyet University, Goztepe Training and Research Hospital, Istanbul, TUR

**Keywords:** arterial inflammation, inflammation, bioactive lipids, lipids, covid-19

## Abstract

Low-density lipoprotein receptor-related protein-1 (LRP1) is an endocytosis receptor that clears inflammatory proteins from circulation. LRP1 has anti-inflammatory effects that bind pro-inflammatory cytokines or ligands. LRP1 has a soluble form (sLRP1) which can be measured in serum. We report sLRP1 levels in hospitalized patients with COVID-19. The first objective of this study is to compare the sLRP1 levels between COVID-19 patients and healthy controls. The second objective is to examine the association between sLRP1 and the clinical outcome of COVID-19.

All patients (20-80 years of age) were evaluated in a hospital using a positive PCR test for SARS‑CoV‑2 between April 1, 2020, and June 1, 2020. Controls (n=59) were selected from healthy subjects. sLRP1 levels were measured in patients from the emergency department (ED), inpatient service (IS), and the intensive care unit (ICU). The study included 180 cases. COVID-19 patients showed significantly lower sLRP1 levels compared to controls (1.43 (1.86) versus 2.27 (1.68) μg/mL, respectively, p<0.001). sLRP1 levels were 1.26 (1.81), 1.37 (1.65), and 1.74 (1.98) μg/mL in patients from ED, IS, and ICU, respectively (p=0.022). Patients who were admitted from ED displayed lower sLRP1 levels compared to those who were discharged (median sLRP1 levels were 0.86 versus 1.7 μg/mL, p=0.045).

COVID-19 patients display significantly lower sLRP1 levels compared to the healthy controls. sLRP1 levels do not show any association with the clinical outcome of COVID-19. This study demonstrates that LRP1 displays a bidirectional course in COVID-19. A low sLRP1 level is a potential risk factor for susceptibility and hospital admission due to COVID-19. Further studies with larger sample sizes and longer follow-ups are needed to understand the long-term effects of novel biomarkers such as sLRP1 on the outcome of COVID-19.

## Introduction

COVID-19 is caused by severe acute respiratory syndrome SARS‑CoV‑2 infection [1]. COVID-19 involves a cascade of events related to vascular risk, inflammation, and thrombosis [1-3]. Severe cases of COVID-19 present with a vicious cycle of increased chemokines, cytokines, and additional proteins. The condition is known as a cytokine storm [2,3]. The presentation of COVID-19 varies from asymptomatic cases or with mild symptoms to serious organ injuries requiring ICU admission [2,3]. The biological basis for the heterogeneity in COVID-19 remains unclear. The length of hospital stay, the need for ICU admission or intubation, and short- and long-term mortality are important clinical outcome points after COVID-19 [4].

COVID-19 is associated with poor outcomes during the first wave of the pandemic [5]. According to a study conducted in New York City, patients with diabetes mellitus (DM) and obesity are more likely to require ICU admission and intubation compared to those without DM or obesity. However, mortality rates are similar between the groups [4]. COVID-19 patients display wide heterogeneity. Therefore, novel biomarkers are needed to understand the determinants of poor outcomes in COVID-19.

Low-density lipoprotein receptor-related protein-1 (LRP1) is a large multifunctional receptor with a variety of structurally diverse ligands [6,7]. As one of the low-density lipoprotein receptor family, LRP1 is also known as the apolipoprotein E receptor [8]. LRP1 is ubiquitously expressed on the cell surface as a large extracellular ligand-binding subunit (515 kDa, α chain) and a short transmembrane subunit (85 kDa, β chain). LRP-1 (CD91) is an endocytosis receptor that clears fibrinolytic system components, complement factors, lipoproteins, and inflammatory cytokines from the circulation [9]. Experimental models demonstrate that soluble or membrane-type LRP1 modulates inflammatory responses [10]. LRP1 modulates inflammatory responses and has anti-inflammatory effects by inhibiting pro-inflammatory factors such as tissue necrosis factor (TNFa), IL-1, IL-6, IL-10, or type 1 interferons [11-13]. Loss of LRP1 results in the dysregulation of inflammation and extracellular proteases [10]. The structure of LRP1 is composed of transmembrane and cytoplasmic domains [14]. LRP1 has a soluble form, which can be detected in the serum [7,15]. sLRP1 (soluble form) is composed of the α-chain and a fragment of the β chain [6,11]. The soluble receptors serve as decoy receptors in the extracellular space and provide a dynamic equilibrium between the membrane receptor and the diverse ligands [15,16]. To the best of our knowledge, the effects of COVID-19 on sLRP1 levels or vice versa remain to be elucidated.

COVID-19 is associated with inflammation. COVID-19 patients display a heterogenous disease course. Novel biomarkers are needed for the early prediction of the disease course to tailor therapy for COVID-19. Since LRP1 has anti-inflammatory effects, we study the sLRP1 levels in hospitalized patients with COVID-19. The first objective of this study is to compare the sLRP1 levels between COVID-19 patients and healthy controls. The second objective is to examine the association between sLRP1 levels and the clinical outcome of COVID-19.

## Materials and methods

This is a cross-sectional study that was conducted in a large tertiary-care COVID-19 center. This study center is located in the center of Istanbul, Turkey. The hospital is a designated COVID-19 care center.

The hospital has 2,000 beds, and the ED receives nearly 400,000 admissions annually. Similar to the Randhawa et al. study conducted in New York City [4], this study was conducted in the megacity of Istanbul with a socioeconomically and culturally diverse population. The study patients were evaluated during the first wave of the COVID-19 pandemic prior to the availability of vaccines and effective therapies. Consecutive patients aged between 20 and 80 years with a positive real-time reverse transcription-polymerase chain reaction (rt-PCR) test for SARS-CoV-2 between April 1, 2020, and June 1, 2020, were included in the study. According to the WHO records until mid-2021, COVID-19 showed two waves of transmission. The first wave occurred in mid-2020 and the second wave in mid-2021. Hence, the patient population comes with unique features. None of the patients were vaccinated at the time of study or had recurrent diseases.

Study population

All patients were seen at the hospital and had COVID-19-related symptoms. In the ED, a healthcare professional collected a nasopharyngeal swab which was positive for SARS-CoV-2. Patients with room air oxygenation < 94 % or respiratory rate > 22 were admitted to the IS. Patients were transferred to the ICU if chest CT revealed severe hypoxia and severe COVID-19-related lung damage. Demographic, clinical, and laboratory parameters were recorded using an institutional electronic medical database. The medical history and hospital course of all rt-PCR-positive patients were mandatorily recorded in the hospital and laboratory information systems. A CT severity score (CT-SS) was used to assess the severity of COVID-19 pneumonia. All patients underwent an 18-month follow-up. The study was conducted according to the 1975 Helsinki Accords and subsequent revisions and approved by the Ethics Committee and Ministry of Health (protocol number 2022-04-09T11_42_37). The committee waived the requirement of informed consent due to the study design and the anonymity of the database. The study conforms to the STROBE Statement in the reports of observational studies. The controls (*n=59*, mean age 47,7±14,3, 15 males, 44 females) were selected from the primary care clinic and were asymptomatic. Sera were collected prior to the COVID-19 pandemic, and none of the controls had clinical suspicion for SARS-CoV-2 infection. COVID-19 testing was not available at the time we selected the controls, either.

PCR test for COVID-19 diagnosis

rt-PCR test kits (Biospeedy COVID-19 RT-qPCR) were utilized to perform the PCR test. Swab samples including viral transport medium were examined using the rt‐qPCR method according to the manufacturer's instructions (Bioeksen). The test kit detects SARS-CoV-2RdRp (RNA-dependent RNA polymerase) single-gene fragment, which is specific to SARS-CoV-2 (FAM Channel). Gene amplification reactions and signal detections were performed using the LightCycler 480 System (Roche Diagnostics GmbH, Mannheim, Germany).

Grading of CT evidence of disease by imaging parameters

All patients were scanned in the supine position with a 16-detector CT scanner (GE Optima CT660 GE Healthcare, Milwaukee, WI). CT scan parameters were as follows: X-ray tube parameters 120 kVp, 300 mAs; rotation time 0.6 second; pitch 1.0; section thickness 5 mm; intersection space 5 mm; and additional reconstruction with a slice thickness of 1.5 mm. We used CT-SS developed by Yang et al. to assess the severity of COVID-19 pneumonia [17]. The lung segments were assessed for the presence and dissemination of ground-glass opacification, crazy-paving pattern, and consolidation. A total of 20 lung segments were scored on chest CT images according to involvement: score 0 (0%), score 1 (less than 50%), or score 2 (equal to or more than 50% of each region). Finally, the sum of all the segment scores was calculated with a range of 0-40. The extent of the lung involvement of the disease was determined by this scoring. The CT-SS determinations were made by a radiologist blinded to the clinical outcome.

Laboratory investigations

Blood samples were obtained immediately after the diagnosis of COVID-19 was confirmed by a positive rt-PCR test and a thorax CT scan compatible with COVID-19 pneumonia. Ferritin, high-sensitive C-reactive protein, and cardiac troponin levels were measured using a Roche Cobas e601 analyzer (Roche Diagnostics Corp., Basel, Switzerland) according to the electrochemiluminescence immunoassay method. Serum uric acid values and lactate dehydrogenase (LDH) activities were measured photometrically using a Roche Cobas c501 analyzer (Roche Diagnostics Corp., Basel, Switzerland). Serum CRP values were measured turbidimetrically using a Roche Cobas c501 analyzer. D-dimers were analyzed using Siemens BCS XP analyzer (Erlangen, Germany) and Innovance D-dimer test kit. Serum sLRP1 concentrations from the same blood sample were measured using the human-specific sLRP1 enzyme-linked immunosorbent assay (Human Low-Density Lipoprotein Receptor-Related Protein 1 ELISA Kit, Cat. No. E 6718Hu, Bioassay Technology Laboratory, Shanghai, China) according to the manufacturer’s instructions (assay sensitivity 0.016 μg/ml, detection range 0.05-15 μg/ml, intra-assay CV < 8%, inter-assay CV < 10%).

Statistical analysis

All data were analyzed using SPSS Statistics v.25 (IBM Corp. Released 2017. IBM SPSS Statistics for Windows, Version 25.0. Armonk, NY: IBM Corp.). The numerical variables were expressed as means (SD) or medians (IQR) and categorical variables as counts and percentages (n, %). The normality of distribution was assessed using the Kolmogorov-Smirnov test, the differences between groups with the chi-square test for categorical variables, and variables with parametric or nonparametric distributions with Student’s t-test or Mann-Whitney U test, respectively. Univariate analyses were performed to evaluate the potential variables associated with the disease severity and mortality presented with an OR and 95% CI. Multivariable logistic regressions were performed to assess the association of clinical characteristics and biochemical results with the study endpoints. The variables with a potential or significant univariate association with the endpoints were included in the multivariate analysis. The statistical significance was defined as a two-sided p-value < 0.05.

## Results

**Figure 1:**
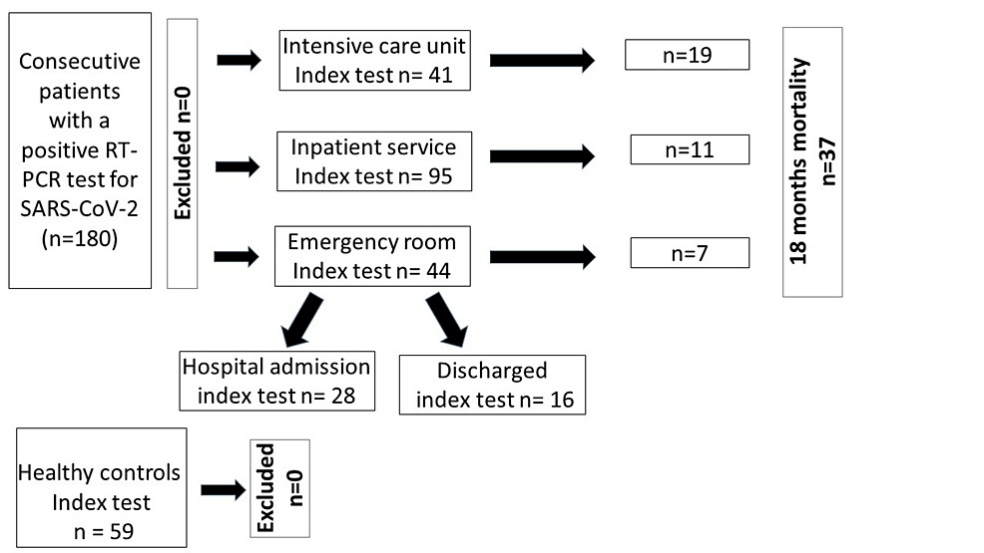
Patients from the ER, IS, and ICU

This study included 180 PCR-positive COVID-19 patients (mean age 64.1±18.6, 91 males, 89 females) and 59 controls (mean age 47.7±14.3, 15 males, 44 females). The COVID-19 patients were from the ER (*n=44*), IS (*n=95*), and ICU (*n=41*) (Figure 1, Table 1).

**Table 1:** Age, laboratory, and clinical characteristics of COVID-19 patients. All patients were seen in the hospital with COVID-19-related symptoms. The patients were divided based on the location of care in the hospital such as the ER, IS, and ICU. +/- standard deviation CT-SS: CT severity score, sLRP1: soluble low-density lipoprotein receptor-related protein-1, LDH: lactate dehydrogenase

	ER (n=44)	IS (n=95)	ISU (n=41)	p-value
Age (years)	59.9 ± 18.9	65.0 ± 18.0	66.2 ± 19.8	0.260
CT-SS	17.4 ± 8.3	14.3 ± 8.5	22.2 ± 8.4	<0.001
18-month mortality (%)	7 (15.9)	11 (11.6)	19 (46.3)	<0.001
sLRP1 (μg/mL)	1.26 (1.81)	1.37 (1.65)	1.74 (1.98)	0.022
Duration of hospital stay (days)	3.5 (5.75)	4 (4)	7 (9)	0.011
LDH (mg/dL)	309 (224)	312 (185)	556 (368)	<0.001
Uric acid (mg/dL)	6.1 ± 2.9	6.2 ± 2.7	7.2 ± 2.9	0.096
High-sensitivity troponin (ng/L)	26 (397)	11 (48)	77 (792)	<0.001
C-reactive protein (μ/mL)	8.2 (13.2)	10.2 (12.4)	16.4 (13.7)	<0.001
D-dimer (μ/mL)	1.28 (1.78)	1.34 (1.39)	2.92 (4.54)	<0.001
Ferritin (mg/dL)	600 (999)	375 (1169)	1504 (4453)	<0.001

**Figure 2:**
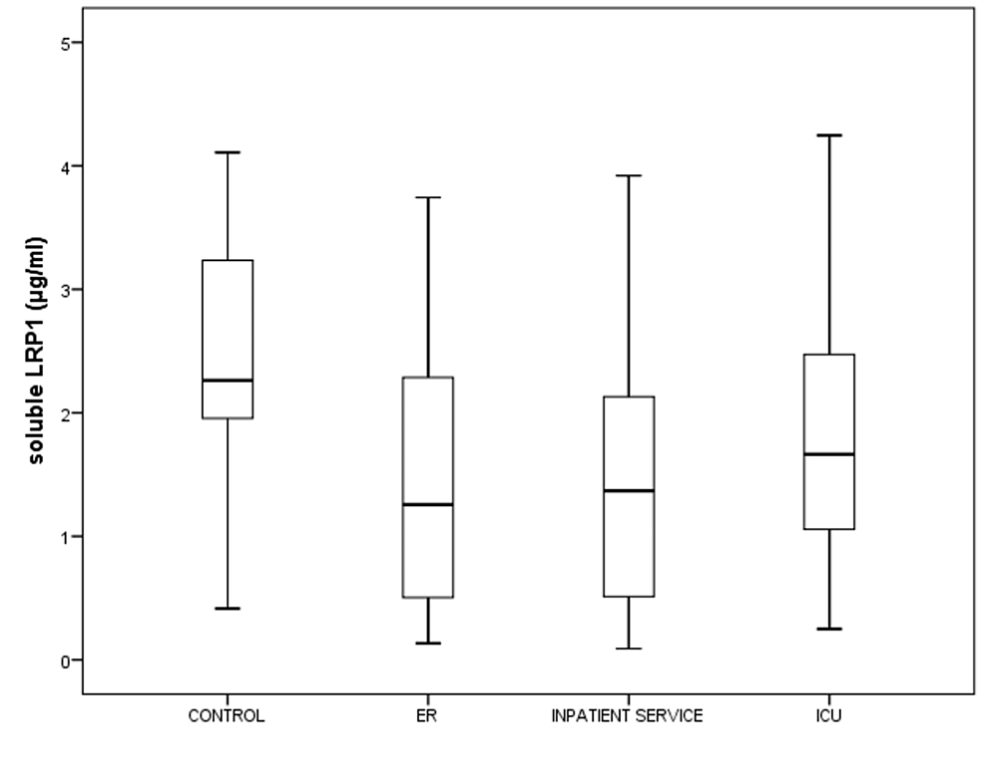
COVID-19 patients displayed significantly lower sLRP1 levels compared to the healthy controls (median sLRP1 levels were 2.27 (1.68) and 1.43 (1.86) μg/mL for controls and COVID-19 patients, sLRP1 were lowest in the ER and increased gradually in the IS and ICU services (p= 0.022), respectively, p<0.001). sLRP1: soluble low-density lipoprotein receptor-related protein-1

The median (IQR) duration of hospital stay was 3.5 (5.75), 4 (4), and 7 (9) days for ER, IS, and ICU patients, respectively (p=0.011). The median CT-SS was 16 (14). Nearly one-third of the patients (36%) displayed severe CT-SS (> 20). Mortality was observed in 37 patients (20.6 %) at 18 months (Figure 1). COVID-19 patients displayed significantly lower sLRP1 levels compared to the healthy controls (1.43 (1.86) μg/mL versus 2.27 (1.68), p < 0.001) (Figure 2). The biomarkers of inflammation and thrombosis were measured in patients from ER, IS, and ICU (Table 1). sLRP1 levels increased with the disease severity and duration of hospital stay. Healthy controls displayed the highest sLRP1 levels. sLRP1 were lowest in the ER and increased gradually in the IS and ICU services (p= 0.022) (Table 1, Figure 2).

**Figure 3:**
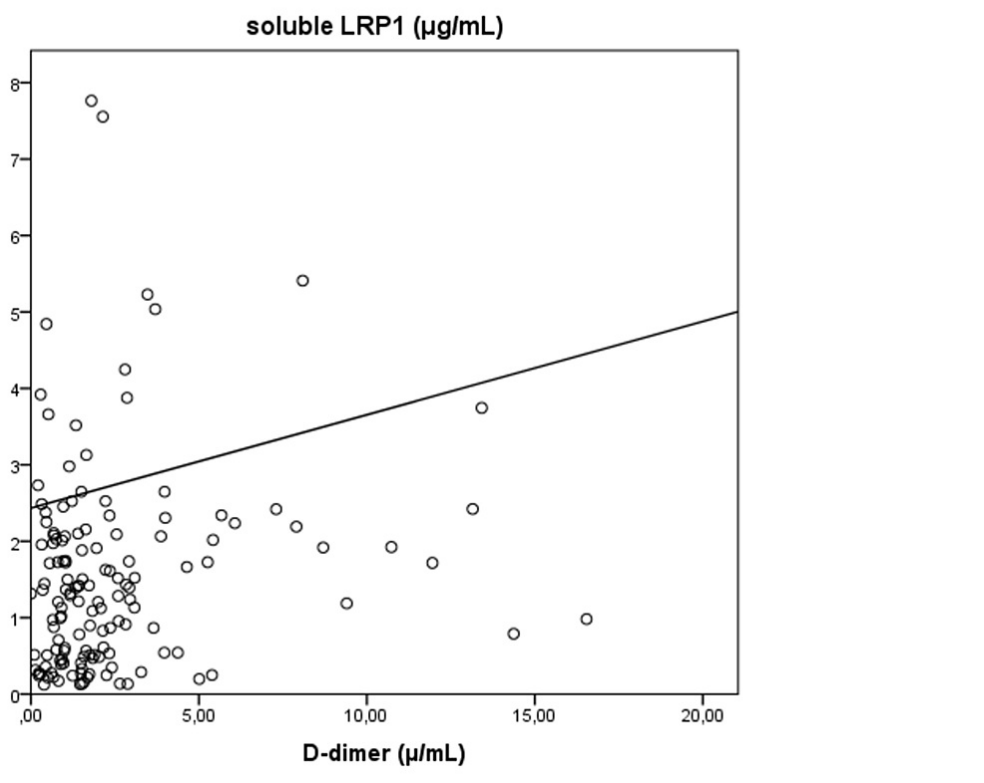
D-dimer and sLRP1 levels correlate positively (Spearman r=0.181, p=0.024). sLRP1: soluble low-density lipoprotein receptor-related protein-1

sLRP1 levels increased with the duration of hospital stay with borderline statistical significance (Spearman r=0.181, p=0.059). D-dimer and sLRP1 levels correlate positively (Spearman r=0.181, p=0.024) (Figure 3).

**Figure 4:**
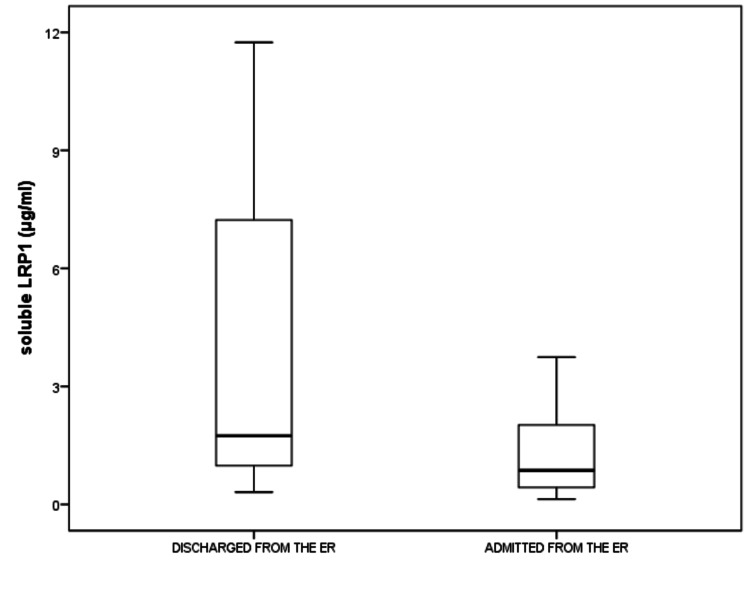
The patients who were admitted to the hospital displayed significantly lower sLRP1 levels compared to the patients who were discharged from the ER (median sLRP1 levels were 0.86 (1.6) versus 1.7 (8.0) μg/mL, p=0.045). sLRP1: soluble low-density lipoprotein receptor-related protein-1

Out of the 44 patients in the ER, 28 patients required hospital admission according to the Ministry of Health protocols. The patients who were admitted to the hospital displayed significantly lower sLRP1 levels compared to the patients who were discharged from the ER (median sLRP1 levels were 0.86 (1.6) versus 1.7 (8.0) μg/mL, p=0.045) (Figure 4). Among the discharged patients from the ER, 1 patient displayed mortality at 18 months. When we compared the COVID-19 patients who were discharged from the ER with the mortality group, there were no differences in sLRP1 levels among the groups (median sLRP1 (1,6 (9,8) versus 1.7 (1.8) μg/mL, p=0.65).

**Figure 5:**
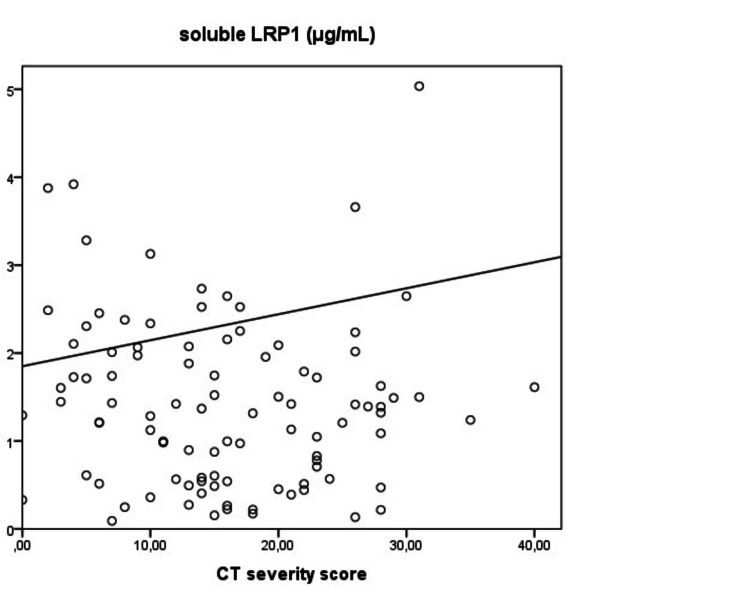
There was a positive correlation between CT-SS and sLRP1 levels in the mortality group (Spearman r=0.467, p=0.039).

Mortality was observed in 37 patients (20.6 %) at 18 months. sLRP1 levels were similar between the mortality group and the survivors (1.78 (1.63) and 1.73 (1.84) μg/mL respectively, p=0.704). The mortality group displayed higher CT-SS compared to the survivors (16.0±8.7 versus 19.9±9.3, p=0.032). There was a positive correlation between CT-SS and sLRP1 levels in the mortality group (Spearman r=0.467, p=0.039) (Figure 5).

According to the univariate analysis, age, CT-SS, LDH, uric acid, high-sensitive troponin, D-dimer, and ferritin levels were associated with a mortality of 18 months (Table 2).

**Table 2:** Univariate and multivariate analyses of predictors of 18-month mortality in COVID-19 patients. Multivariate logistic regression analysis was performed to find the predictors of 18-month mortality. High-sensitive cardiac troponin was the only parameter that was associated with 18-month mortality. CT-SS: CT severity score, sLRP1: soluble low-density lipoprotein receptor-related protein-1, LDH: lactate dehydrogenase

Variable	Univariate			Multivariate		
	OR	95% CI	P	OR	95% CI	P
Age	1.064	1.037-1.093	<0.001	1.063	0.985-1.147	0.114
Gender	0.630	0.312-1.274	0.199			
CT-SS	1.051	1.003-1.100	0.035	1.082	0.947-1.237	0.246
sLRP1 (μg/ml)	0.960	0.893-1.032	0.271			
LDH (mg/dl)	1.005	1.003-1.007	<0.001	1.004	0.999-1.008	0.137
Uric acid (mg/dl)	1.499	1.271-1.767	<0.001	1.152	0.775-1.712	0.484
High-sensitive troponin (ng/L)	1.012	1.007-1.018	<0.001	1.011	1.001-1.021	0.032
C-reactive protein (mg/dl)	1.007	0.996-1.017	0.212			
D-dimer (mg/dl)	1.992	1.517-2.616	<0.001	1.697	0.911-3.163	0.096
Ferritin (mg/dl)	1.000	1.000-1.000	0.002	1.000	1.000-1.000	0.890

A multivariate logistic regression analysis was conducted to determine the predictors of the 18-month mortality. High-sensitive cardiac troponin was the only parameter that was associated with mortality in the multivariate logistic regression analysis (Table 2). sLRP1 was not associated with 18-month mortality in the uni- or multivariate analysis (Table 2).

## Discussion

The study included COVID-19 patients during the first wave of the pandemic. LRP1 is a poorly understood multifunctional receptor with a variety of structurally diverse ligands [6,7]. The first objective of this study is to compare the sLRP1 levels between COVID-19 patients and healthy controls. COVID-19 patients display significantly lower sLRP1 levels compared to the healthy controls. The patients who required admission to the hospital displayed significantly lower sLRP1 levels compared to those who were discharged from the ED. The second objective is to examine the association between sLRP1 levels and the clinical outcome of COVID-19. sLRP1 levels vary widely among COVID-19 patients and do not seem to predict the clinical outcome points or survival after COVID-19. The results indicate that, unlike other inflammatory biomarkers of COVID-19 (i.e., hsTrop, C-reactive protein, D-dimer, and ferritin), sLRP1 displays a biphasic response in COVID-19. Low sLRP1 levels are observed throughout the course of COVID-19. In hospitalized patients, sLRP1 levels increase with the D-dimer level and duration and severity of COVID-19 but still remain to be lower than the healthy controls.

Our observations support prior preclinical studies on LRP1. Experimental models indicate that LRP1 is a potential defense mechanism in acute viral infections and related organ injuries [18-20]. LRP1 expression increases after *Herpesviridae* infection [18]. Eliminating LRP1 by siRNA knockdown increases the infectious virus yield. LRP1 restricts human cytomegalovirus infectivity by controlling the availability of cholesterol for the virion envelope, and increased LRP1 expression is likely a defense response to infection [18].

Similar to Sars-CoV-2, the Rift Valley fever virus (RVFV) is a zoonotic pathogen. The RVFV entry relies on host entry factors which remain to be elucidated. According to a study, RVFV glycoprotein directly binds to LRP1. The exogenous addition of anti-LRP1 antibodies neutralizes RVFV infection in taxonomically diverse cell lines [19]. Our finding of a low sLRP1 in COVID-19 is also supported by prior experimental studies with the dengue virus (DenV) [20]. DenV infection modifies LRP1 protein expression to maintain host-derived intracellular cholesterol. Viral infection reduces LRP-1 protein expression, and dsRNA knockdown of LRP1 increases intracellular cholesterol and DenV viral RNA.

Similarly, the biological and clinical effects of LRP1 or sLRP1 levels in chronic and complex diseases remain to be elucidated. A prior clinical study reported that lower sLRP1 levels are observed in diabetic patients with retinopathy compared to healthy controls [21]. Other studies indicated that sLRP1 is associated with the presence of epicardial fat, hyperlipidemia, or atherosclerosis [16,22]. The effects of LRP1 on chronic complex diseases do not appear to be through altering levels and function of serum lipoproteins [21].

This study was conducted in a large COVID-19 center during the first wave of the pandemic. Since the study period, the COVID-19 phenotype has changed significantly through vaccines and novel variants. The study population was unique as none of the patients were vaccinated at the time of study or had recurrent diseases. None of the patients had the time to complete full-dose vaccination at the time of follow-up. Novel variants, such as the delta variant, had not yet reached global circulation at the time of the study. The first variants were dominant throughout the world. Therefore, the diagnostic and prognostic forecast value of biomarkers will also change according to the globally dominant variants at the time of the studies. COVID-19 in Turkey during the first wave was associated with high mortality [5]. The study population similarly showed high mortality at 18 months. The transformation of SARS-CoV-2 in a continuum, novel variants with breakthrough infections, and changing phenotype of chronic complex diseases are future concerns.

Highly sensitive cardiac troponin was the strongest parameter in the prediction of 18-month mortality. Our findings add to the nascent body of literature on cardiac complications of COVID-19. Despite extensive efforts to treat the pulmonary component of COVID-19, data surrounding the cardiac complications associated with the virus are growing [23,24]. A previous study reported that in big cities with high COVID‐19 cases, defibrillator shocks increase during the COVID‐19 outbreak [24]. Future studies are needed to elucidate the long-term effects of COVID-19 on cardiac disease phenotype. 

LRP1 is cardioprotective due to the activation of the reperfusion injury salvage kinase pathway after ischemia-reperfusion [25]. Although the underlying mechanisms of action are still poorly described, sLRP1 level alterations can be temporary in the disease course of COVID-19. Understanding the molecular mechanisms of organ injury in COVID-19 is crucial for the development of potential therapeutic targets. This study included COVID-19 cases during the first wave of the pandemic between April and June 2020. None of the study patients were vaccinated and contracted a recurrent infection of SARS-CoV-2. Follow-up studies of cases during the first wave can elucidate the biology of poor outcomes in COVID-19.

This study comes with limitations. The study population and timing in the disease course affect the performance of the biomarker in post-infectious diseases. The distribution and functional characteristics of sLRP-1 levels in chronic complex diseases still remain controversial. The lower levels of sLRP-1 in COVID-19 patients compared to healthy controls can, therefore, increase our understanding of LRP1. Variants of the virus, availability of vaccines, health system organization, and local and global changes in the disease phenotype all affect the COVID-19 outcome. The association studies between COVID-19 outcomes and biomarker levels commonly display reverse causality. Patients with low sLRP1 are likely to have morbidities such as vascular diseases. The control group is small and consists of younger subjects compared to the COVID-19 cases. The patients were from the ED, IS, and ICU. The factors that are affecting the clinical outcome in adults and pediatric age groups differ in COVID-19 [26,27]. The mortality is high in the much older population (≥80 years) during the first wave [27}. Further research is required to study the differences between younger and much older populations [26,27]. This is a cross-sectional study with a limited number of cases. We do not know if COVID-19 patients have low sLRP1 levels at baseline or if COVID-19-induced inflammation reduces sLRP1 levels. The confounding effects of genetic and environmental risk factors cannot be excluded.

## Conclusions

COVID-19 patients display significantly lower sLRP1 levels compared to the controls. sLRP1 levels do not show any association with the clinical outcome of COVID-19. This study demonstrates that LRP1 displays a bidirectional course in COVID-19. Low sLRP1 level is a potential risk factor for susceptibility and hospital admission due to COVID-19. The transformation of SARS-CoV-2 in a continuum, novel variants with breakthrough infections, and changing phenotype of chronic complex diseases are future concerns. Further studies with larger sample sizes and longer follow-ups are needed to understand the long-term effects of novel biomarkers, such as sLRP1, on the outcome of COVID-19.
